# College and Hospital Note

**Published:** 1896-12

**Authors:** 


### College and Hospital Note.

The faculty of Albany Medical College is to be congratulated upon receiving the donation of a building for pathological and bacteriological work, the gift of Mr. Matthew W. Bender, of Albany, to be known as the Bender hygienic laboratory. The building was formally dedicated, Tuesday evening, October 27, 1896, to which we acknowledge the receipt of an invitation. The ceremonies consisted in part of the formal presentation of the building to the board of trustees in the name of the donor by Harry H. Bender, Esq., an address on the history of the building by Dr. George E. Gorham, reception of the building on behalf of the board of trustees by Dr. A. Vander Veer, and an address commemorative of the event and the importance of pathological and bacteriological study by Dr. A. Jacobi, of New York.

